# Influence of ovarian cancer type I and type II microenvironment on the phenotype and function of monocyte-derived dendritic cells

**DOI:** 10.1007/s12094-017-1686-2

**Published:** 2017-06-06

**Authors:** J. Surówka, I. Wertel, K. Okła, W. Bednarek, R. Tarkowski, J. Kotarski

**Affiliations:** I Chair and Department of Oncological Gynaecology and Gynaecology, Medical University of Lublin, Al. Racławickie 1, 20-059 Lublin, Poland

**Keywords:** Ovarian cancer, Dendritic cells, Regulatory T cells, Peritoneal fluid, Cell cultures

## Abstract

**PURPOSE:**

The aim of this study was to evaluate the influence of ovarian cancer cell lysates isolated from type I or type II ovarian cancer (OC) on the phenotype of monocyte-derived dendritic cells (Mo-DCs) and the cytokine profile. We also determined whether the Mo-DCs and tumor microenvironment, reflected by peritoneal fluid (PF) from type I or II ovarian cancer, could promote regulatory T cell (Tregs) differentiation from naive CD4^+^ lymphocytes in vitro.

**RESULTS:**

Our results show a significant role of the ovarian cancer microenvironment reflected by PF from type I or II OC in the inhibition of the DC differentiation process. Interestingly, the percentage of cells co-expressing CD45 and CD14 antigens in the cultures stimulated with PF from both type I and type II OC was higher than in the control. Furthermore, the percentage of cells expressing CD1a, i.e., a marker of immature DCs, was significantly reduced in the cultures stimulated with PF from type I and type II OC. The results obtained show that ovarian cancer type II lysates induce differentiation of monocytes into macrophage-like cells with a CD1a^+^/HLA-DR^+^/CD83^−^ phenotype and significantly higher CD86/HLA-DR expression. We show that ovarian cancer type II Mo-DCs are able to prevent an immune response by release of IL-10, whereas OC type I Mo-DCs can promote the generation of Tregs.

**CONCLUSIONS:**

We demonstrate that each type of ovarian cancer can induce a unique phenotype of DCs and differentiation of Tregs, both associated with immune-suppressive function, which may be an obstacle while developing effective anticancer dendritic cell vaccination.

## Introduction

Ovarian cancer (OC) is one of the most lethal gynecological malignancies with 239,000 new cases diagnosed worldwide only in 2012 and the number of deaths estimated at over 140,000 [1]. The insufficient number of effective therapeutic tools in OC prompts a search for new approaches, such as dendritic cell (DC)-based immunotherapy [2].

According to the classification proposed in 2004 by Kurman and Shih [3], ovarian cancer can be divided into two subtypes with different genetic and clinicopathologic features: type I and type II. Type I tumors include low-grade serous, mucinous, endometrioid, and clear cell carcinomas. They are less aggressive, with a low grade and better prognosis. In contrast, type II tumors include mainly high-grade serous and undifferentiated carcinomas. Ovarian cancer in this group is diagnosed at a high stage of the disease; they develop rapidly and are highly aggressive [4]. It is well known that the immune system is imbalanced in women with ovarian cancer [5]. However, the interactions between the immune and tumor cells in the different types of OC patients remain unclarified. We hypothesized that different types of OC may influence the phenotype and function of monocyte-derived dendritic cells (Mo-DCs).

Dendritic cells play a crucial role in initiating and controlling innate and adaptive immunity. They possess a unique ability to activate naive T cells [6]. This exclusive feature places DCs as a promising new anti-tumor therapy source. A few clinical trials have tested the efficacy of DCs loaded with whole tumor lysates in cancer therapies, some with very promising results [7–10]. However, although immunological responses are observed in some OC patients, clinical responses are only detected in a minority of them [5].

It was shown that DCs are a highly heterogeneous population of cells with different features. Depending on their subpopulation and maturation level, they can either activate immune response or induce immunotolerance [6]. Furthermore, the OC microenvironment is enriched with factors such as cytokines and angiogenic factors, which can alter the phenotype and functions of DCs [5]. Apart from that, tumor cells can recruit immature myeloid DCs that induce differentiation of regulatory T cells (Tregs) [11]. Tregs not only maintain immune tolerance to self-antigens but also suppress the activation of the immune system [5]. Increased percentages of Tregs were detected in peripheral blood (PB) and tumor microenvironment, and were correlated with a poor prognosis and shorter survival times of cancer patients [12, 13]. Tregs can accumulate in the tumor microenvironment via several mechanisms including recruitment, expansion, and conversion. Their conversion from non-suppressive T cells has been mainly studied in murine models and this is to be confirmed in human cancer models. An understanding of the patient immune system and its ability to mount anti-tumor immune responses to cancer is a critical component of immunotherapy design.

The aim of this study was to evaluate the influence of autologous ovarian cancer cell lysates (OCL) isolated from type I or type II OC on the Mo-DCs phenotype and the cytokine profile. Another goal of the study was to adjudicate the influence of the tumor microenvironment reflected by peritoneal fluid (PF) on the Mo-DCs phenotype. Our next objective was to determine whether Mo-DCs and PF from type I or II ovarian cancer can promote differentiation of Tregs from naive CD4^+^ lymphocytes in vitro.

## Materials and methods

Peripheral blood, PF, and ovarian cancer tissue were obtained from 17 patients operated in the I Chair and Department of Oncological Gynaecology and Gynaecology (Lublin, Poland). The patients were assigned into two categories: type I (*n* = 9) and type II (*n* = 8) ovarian cancer, according to the Kurman and Shih classification [3, 4]. Patients’ clinicopathologic characteristics are presented in Table 1. Exclusion criteria for participation in the study included a history of previous malignances, autoimmune and allergic diseases, and any signs of an ongoing infection. All the patients provided a written informed consent for the study allowing ex vivo experimentation, which was approved by the Bioethics Committee of the Medical University of Lublin.

**Table 1:** Patients’ characteristics

	*N* (%)
FIGO stage	
1	2 (11.76)
2	7 (41.18)
3	5 (29.41)
4	3 (17.65)
Grade	
G2	9 (52.94)
G3	8 (47.06)
Histological type of CO	
Serous	3 (17.65)
Endometrioid	8 (47.06)
Mucinous	4 (23.53)
Undifferentiated	2 (11.76)
Type of OC according to Kurman’s classification	
1	9 (52.94)
2	8 (47.06)

Other features	Median (min–max)
Age	55 (22–80)

Peripheral blood samples (20 ml) were collected in sterile heparinized tubes the day before the surgical procedures were done. Peritoneal fluid and tumor tissue were collected at the time of surgery. Only PF without signs of hemolysis was accepted for the study. Tumor tissue samples were collected from the primary tumor site and divided into two parts: one part was sent for histopathological verification and the other part was used for in vitro studies. Autologous OCL were isolated from type I or type II tumors and prepared by a quintuple cycle of rapid freeze (−80 °C) and thaw (37 °C) of the cell suspension in 1 ml of RPMI 1640 without phenol red (PanBiotech, Germany). Next, the samples were centrifuged at 700×*g* for 5 min; cell supernatants were collected and filtered through 0.20 μm microfilters. The density of the lysates was measured spectrophotometrically.

### Generation of dendritic cells

**Fig. 1:**
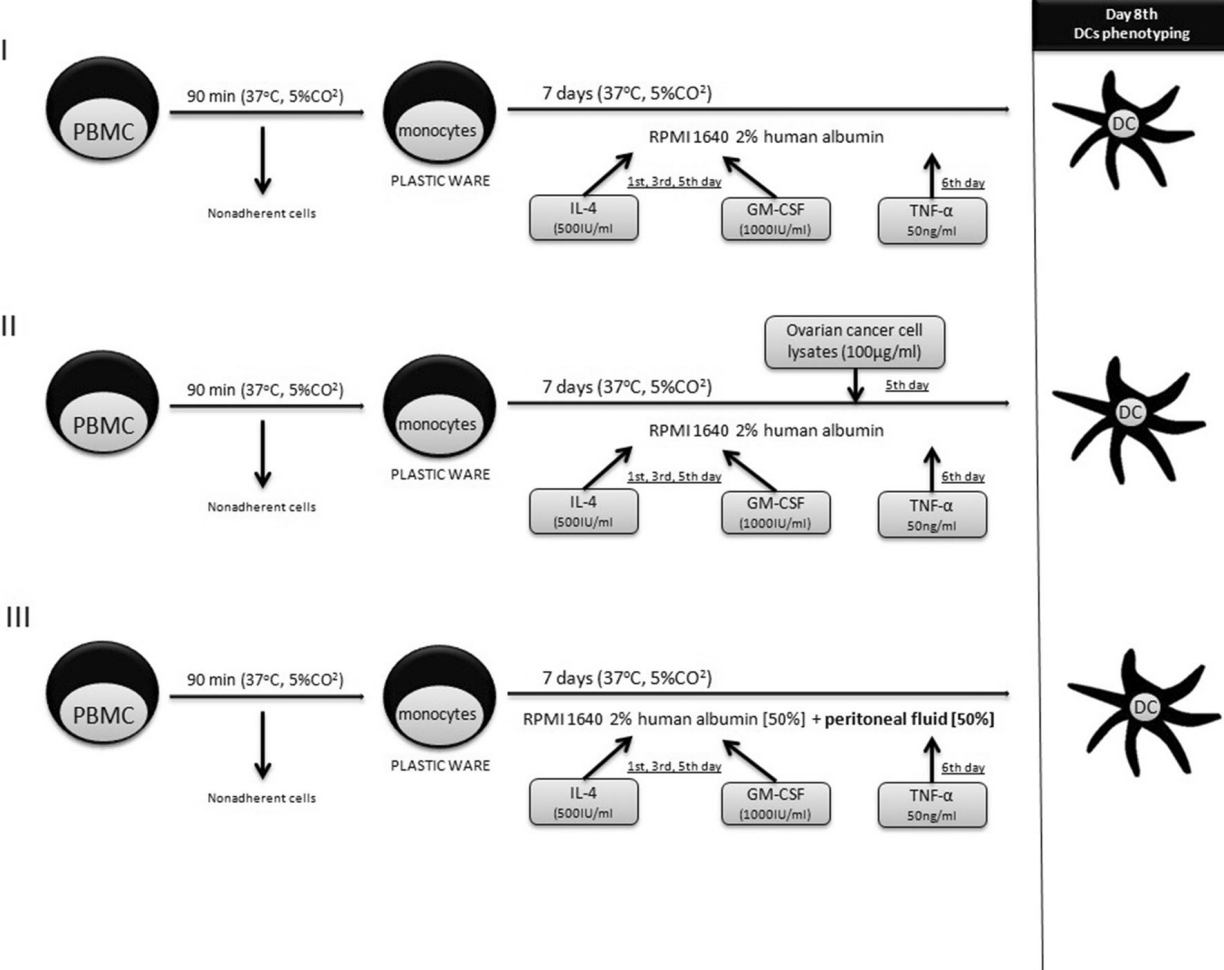
Generation of dendritic cells

Peripheral blood mononuclear cells (PBMCs) were isolated from PB by density gradient centrifugation using Lymphoprep (STEMCELL Technologies, Canada). Interphase cells were aspirated, washed twice in PBS without Ca^2+^ and Mg^2+^, and resuspended in RPMI 1640 enriched with 2% human albumin (Baxter, Vienna, Austria). Monocytes were isolated by adherence to the plastic surface and incubated for 90 min in the standard conditions (37 °C, 5% CO_2_ atmosphere). Nonadherent cells were removed by an intense wash with PBS without Ca^2+^ and Mg^2+^ and cultured for 7 days in RPMI 1640 enriched with 2% human albumin. On the 1st, 3rd, and 5th day, the cultures were supplemented with rhIL-4 (500 IU/ml) and rhGM-CSF (1000 IU/ml). OCL (100 μg protein per ml) from type I or type II tumors were added to the culture on the 5th day. On the 6th day, rhTNF-α (50 ng/ml) was added to induce DC maturation. Dendritic cells were generated in three combinations: control DC culture (I), DCs loaded with OCL from type I or type II OC (II), and DCs stimulated with PF (III). The phenotype of the generated DCs was assessed by flow cytometry after 7 days of the culture (Fig. 1).

### Dendritic cell phenotyping in patients with type I and type II ovarian cancer

The phenotype of the generated DCs was assessed using flow cytometry (BD FACS Canto I. USA). Surface antigen (Ag) staining was performed using mouse anti-human monoclonal antibodies (mAbs) against CD14, CD45, CD1a, CD83, HLA-DR, CD80, and CD86 (BD Biosciences, USA). Based on Ag expression, DCs were identified as: immature DCs: CD1a^+^/HLA-DR^+^/CD83^−^, semi-mature DCs: CD1a^+^/HLA-DR^+^/CD83^+^, and mature DCs: CD83^+^/HLA-DR^+^/CD1a^−^.

### Evaluation of secretory function of Mo-DCs in type I and type II ovarian cancer patients

The IL-10, IL-12, and TNF-α levels were assessed in the supernatants from the Mo-DC cultures using ELISA kits (R&D Systems, USA) and TGF-β1 (eBioscience, Austria). The supernatants used for the detection of cytokines were stored frozen until analysis. The concentration of IL-10 was measured using a Human IL-10 Quantikine HS ELISA kit with a sensitivity of 0.09 pg/ml, IL-12 using a Human IL-12 with a sensitivity of 0.5 pg/ml, and TNF-α using a Human TNF-α Quantikine HS ELISA kit with a sensitivity of 0.106 pg/ml. The concentration of TGF-β1 was measured using a Human TGF-β1 Platinum ELISA kit with a sensitivity of 8.6 pg/ml. All samples were analyzed in duplicate.

### Evaluation of Tregs percentage

To assess whether the generated Mo-DCs and factors present in the ovarian cancer microenvironment can induce differentiation of Tregs, lymphocytes were cultured for 7 days in the following combinations: control culture of lymphocytes, lymphocytes stimulated with autologous PF (diluted 1:1 with RPMI 1640 Cell Culture Medium), and coculture of lymphocytes with autologous OC type I or II lysate-pulsed DCs. The percentage of CD4^+^CD25^high^/FoxP3^+^ cells in the whole CD4^+^ T cell population was assessed using a Human Treg Flow™ Kit (Biolegend, USA) and flow cytometry. The procedure was performed according to the manufacturer’s protocol as described by us previously [14].

### Statistical analysis

Statistical analysis was performed using Statistica 10.0 PL software. The Wilcoxon Test was used to determine the differences between the analyzed cultures. The Mann–Whitney *U* test was applied for statistical comparison of the results between the two studied groups. The results are presented as a median (Med), minimum (Min), and maximum (Max). *p* value less than 0.05 was considered statistically significant.

## Results

### Analysis of CD45/CD14 expression in the Mo-DCs generated with OCL and PF from type I or type II tumors

The percentage of cells expressing CD45/CD14 antigens was higher in the cultures stimulated with both OCL and PF from type I and type II tumors, but the differences did not reach statistical significance (*p* > 0.05) (Table 2).

**Table 2:** Expression of chosen Mo-DC antigens in the cultures generated with OCL and PF from type I or type II tumors

	Type I OC	Type II OC
Ovarian cancer lysates		
CD45^+^/CD14^+^	↑ NS	↑ NS
CD1a^+^/HLA-DR^+^/CD83^−^	↓ NS	↓ *p* = 0.03
CD83^+^/HLA-DR^+^/CD1a^−^	↑ NS	↑ NS
CD1a^+^/HLA-DR^+^/CD83^+^	↓ NS	↓ NS
CD80^+^/HLA-DR^+^	↓ NS	↓ NS
CD86^+^/HLA-DR^+^	↑ *p* = 0.04	↑ *p* = 0.01
CD80^+^CD86^+^HLA-DR^+^	↑ NS	↑ NS
Peritoneal fluid		
CD45^+^/CD14^+^	↑ NS	↑ NS
CD1a^+^/HLA-DR^+^/CD83^−^	↓ *p* = 0.02	↓ *p* = 0.01
CD83^+^/HLA-DR^+^/CD1a^−^	↓ NS	↓ NS
CD1a^+^/HLA-DR^+^/CD83^+^	↓ NS	↓ NS
CD80^+^/HLA-DR^+^	↓ NS	↓ NS
CD86^+^/HLA-DR^+^	↓ *p* = 0.02	↑ NS
CD80^+^CD86^+^HLA-DR^+^	↓ NS	↓ *p* = 0.01

### Analysis of immature (CD1a) and mature (CD83) DC markers in the cultures generated with OCL and PF from type I or type II tumors

**Fig. 2:**
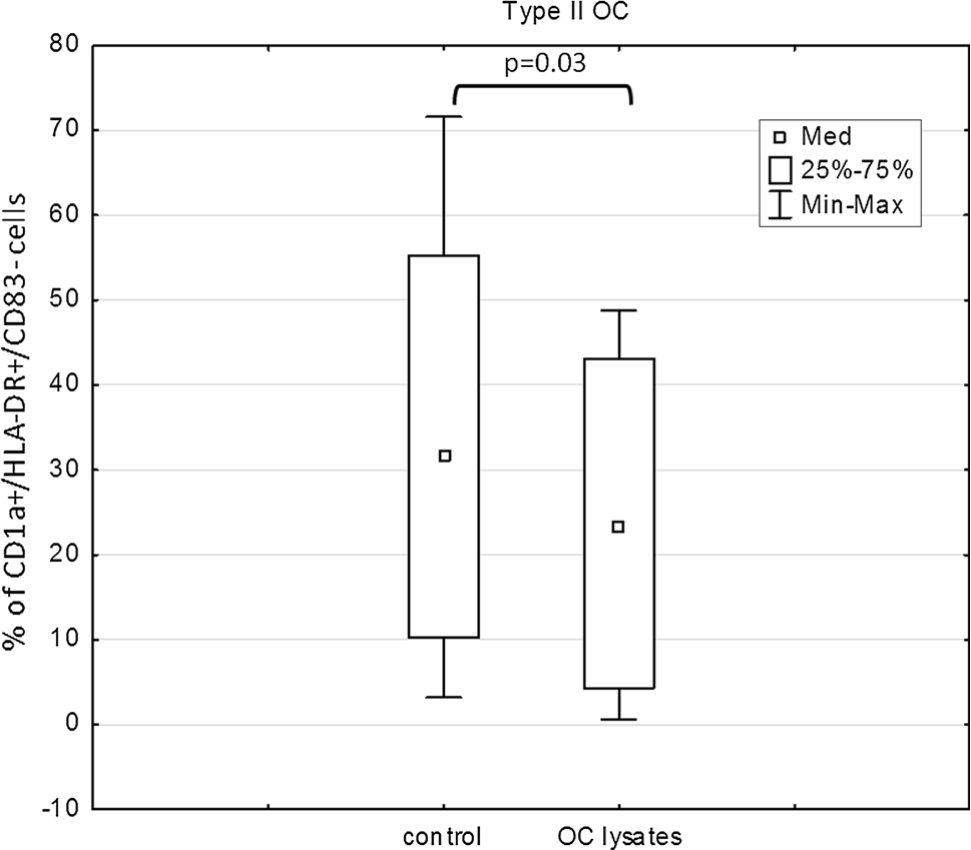
Expression of CD1a/HLA-DR antigens in the Mo-DC cultures generated with OCL

**Fig. 3:**
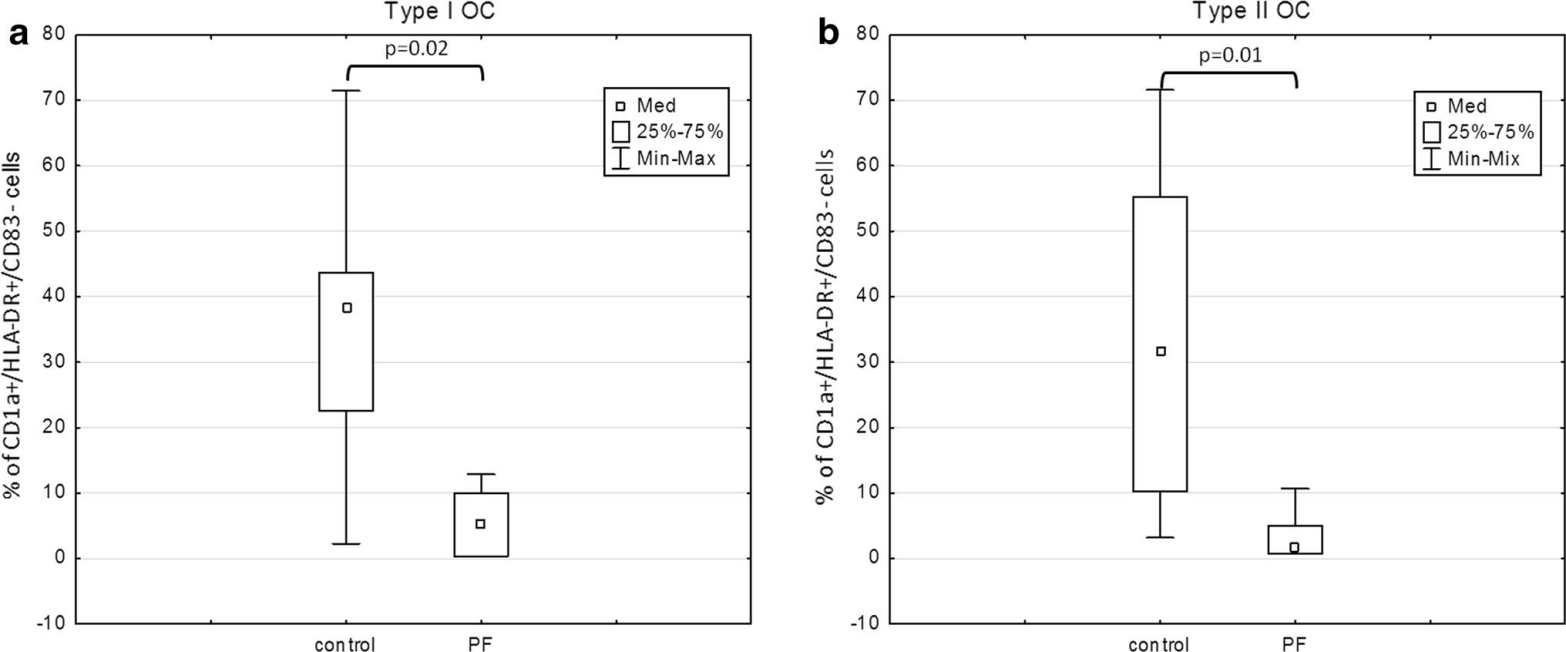
Expression of CD1a/HLA-DR antigens in Mo-DC cultures generated with PF from type I or type II tumors

The percentage of immature CD1a^+^/HLA-DR^+^/CD83^−^ cells was significantly lower (*p* = 0.03) in the Mo-DC cultures loaded with OCL from type II tumors compared with the control (Fig. 2). Moreover, the percentage of cells expressing CD1a/HLA-DR antigens was significantly reduced under the influence of PF in both type I (*p* = 0.02) and type II tumors (*p* = 0.01) (Fig. 3a, b).

The percentage of mature (CD83^+^/HLA-DR^+^/CD1a^−^) DCs was higher in the OCL type I and type II loaded DCs in relation to the control, while the percentage of semi-mature (CD1a^+^/HLA-DR^+^/CD83^+^) DCs was lower in the OCL type I and type II loaded DCs; however, the differences were not significant (*p* > 0.05). The percentage of mature and semi-mature DCs was lower in Mo-DCs generated with PF from type I and type II OC patients; however, these differences did not reach statistical significance (*p* > 0.05) (Table 2).

### Analysis of the co-stimulatory (CD80/CD86/HLA-DR) molecule expression in cultures generated with OCL and PF from type I or type II tumors

**Fig. 4:**
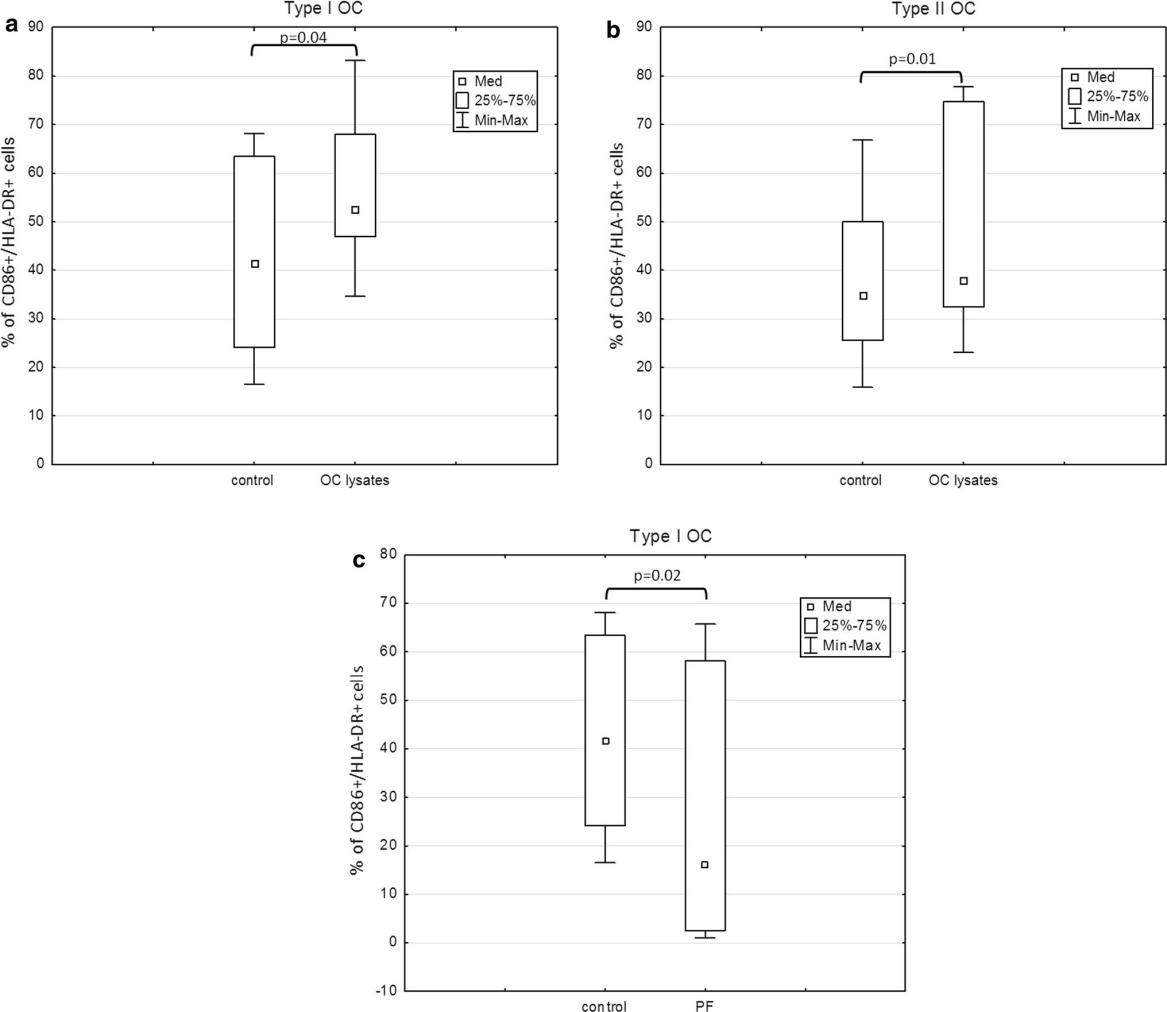
Expression of CD86/HLA-DR molecules in cultures generated with OCL and PF from type I or type II tumors

**Fig. 5:**
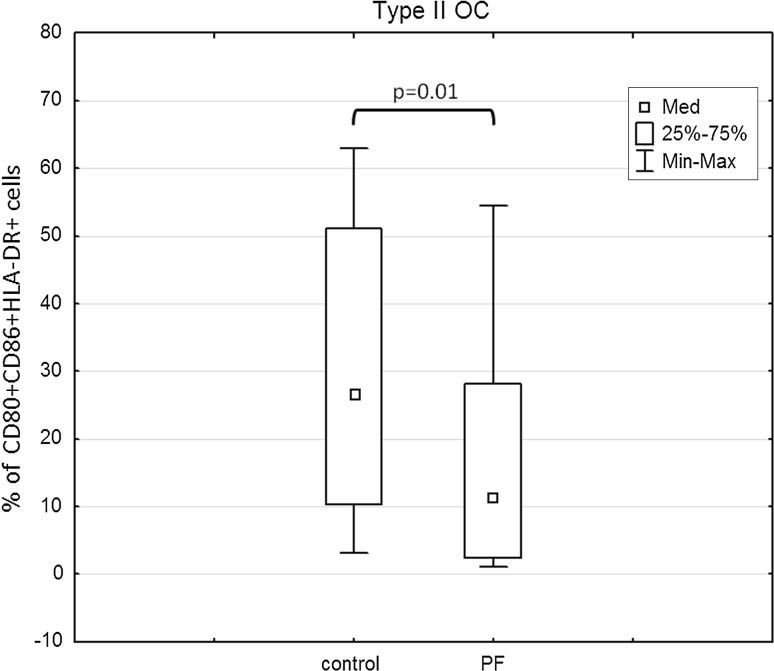
Expression of co-stimulatory (CD80/CD86/HLA-DR) molecules in cultures generated with PF from type II tumors

The percentages of CD86^+^/HLA-DR^+^ cells were significantly higher in Mo-DC cultures stimulated with both OCL from type I (*p* = 0.04) and type II (*p* = 0.01) tumors in relation to the control culture (Fig. 4a, b). In contrast, the percentage of CD86^+^/HLA-DR^+^ was significantly lower in Mo-DCs generated with PF from type I OC (*p* = 0.02) in relation to the control (Fig. 4c). Moreover, the percentage of CD80^+^CD86^+^HLA-DR^+^ cells in Mo-DCs generated with PF from type II OC patients was significantly lower (*p* = 0.01) in relation to the control (Fig. 5). The percentages of CD80^+^/HLA-DR^+^ cells were lower in Mo-DC cultures stimulated with both OCL from type I or type II tumors in relation to the control and were reduced under the influence of PF; however, the differences were not significant (*p* > 0.05).

### Mo-DCs generated from type II ovarian cancer produce higher amounts of IL-10

The second objective of the study consisted in characterization of the cytokine profiles of monocyte-derived dendritic cells from type I and type II OC. Mo-DCs generated from type II OC secreted significantly higher (*p* = 0.04) levels of immunosuppressive IL-10 compared to the cells sampled from patients with type I OC (Table 3). There were no significant differences in the levels of IL-12, TGF-β1, and TNF-α between the Mo-DC cultures generated from type I and type II OC patients (*p* > 0.05).

**Table 3:** Cytokine profiles of Mo-DCs from type I and type II OC

Cytokine	Type I OC Med (min–max)	Type II OC Med (min–max)
IL-10	0.84 (0.02–2.42)	2.42 (0.61–11.02)*
IL-12	0.13 (0.07–0.27)	0.13 (0.06–0.36)
TGF-β	667.83 (136.66–1711.90)	589.68 (143.46–1780.70)
TNF-α	58.11 (54.73–62.84)	59.56 (53.33–62.60)

* *p* = 0.04

### Both peritoneal fluid and Mo-DCs can induce differentiation of Tregs from naive CD4^+^ T cells in vitro

**Fig. 6:**
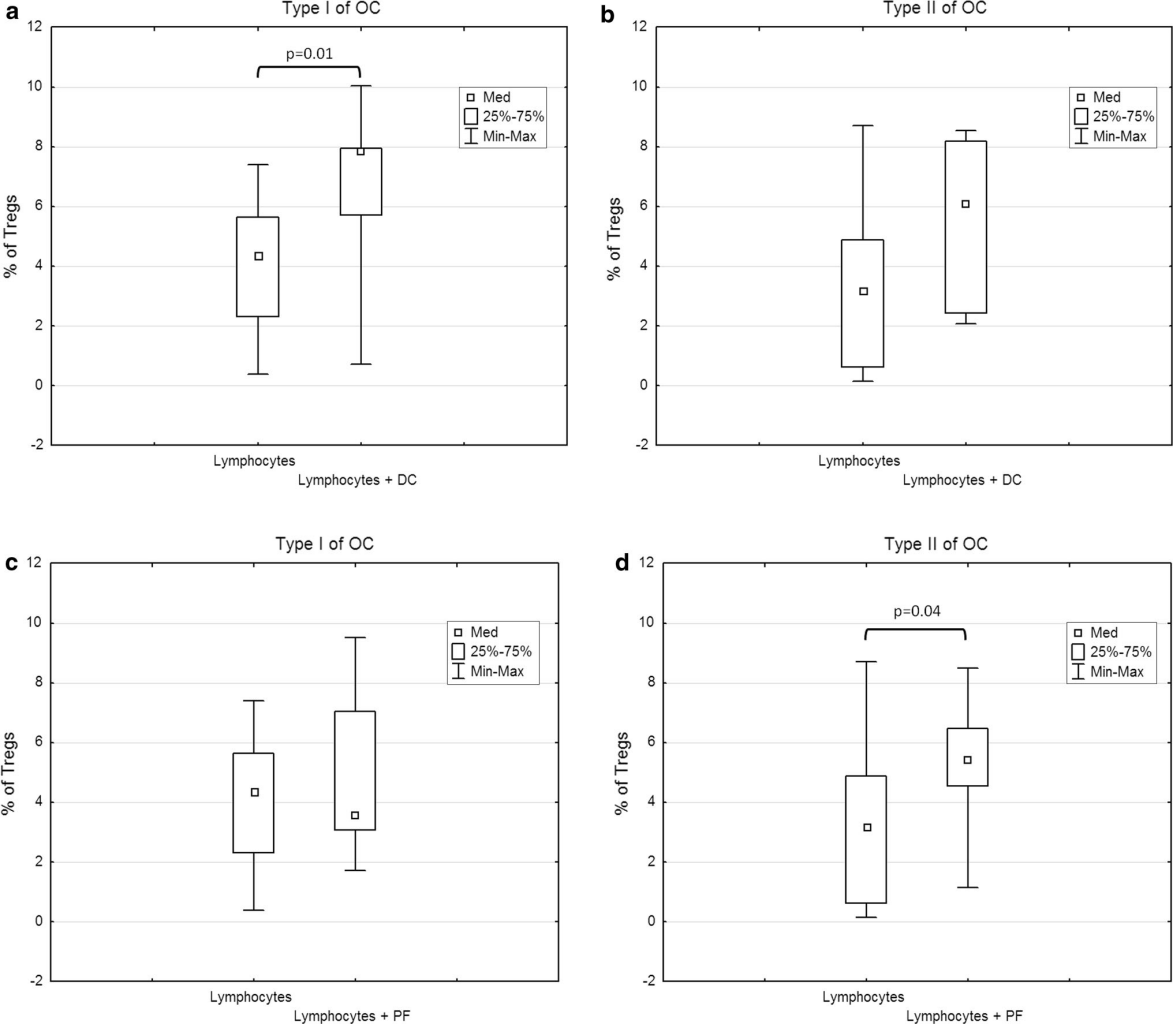
Percentage of Tregs in cultures of autologous T cells with Mo-DCs and PF from type I or type II tumors

Next, we investigated whether the autologous peritoneal fluid or Mo-DCs loaded with OCL are able to generate differentiation of Tregs in vitro. Interestingly, compared with the control, a significantly increased (*p* = 0.01) percentage of Tregs was detected in the co-cultures of DCs with autologous lymphocytes in type I OC. We did not observe such a difference in type II OC (*p* > 0.05) (Fig. 6a, b). However, in type II OC, the percentage of Tregs in the cultures stimulated with autologous PF was significantly higher (*p* = 0.04) compared to the control (Fig. 6c, d). There was no statistically significant difference in the percentages of Tregs between the control cultures and cultures stimulated with PF from type I OC (*p* > 0.05). This may suggest a different mechanism triggering Tregs differentiation in type I and type II OC patients.

## Discussion

Dendritic cell-based vaccines may be a potent tool for immunotherapy of ovarian cancer. Particularly, whole tumor cell lysates can provide a wide range of cancer antigens exclusively for OC. Nevertheless, although immunological responses are observed in some patients, clinical responses are only detected in a minority of them [3].

It is well established that during the cultures, monocytes supplemented with rhIL4, rhGMCS, and rhTNF-α lose the expression of CD14, i.e., a marker specific for monocytes, and acquire markers typical for DCs. This is in agreement with our previous studies, where we have described that monocytes isolated from PB of OC patients may be differentiated ex vivo into DCs [15]. Interestingly, in the cultures stimulated with PF from type I and type II OC, the percentage of cells co-expressing CD45 and CD14 antigens was higher than in the control DC cultures. These results may indicate a significant role of the ovarian cancer microenvironment in the inhibition of the DC differentiation process.

Here, we have demonstrated that, in the cultures stimulated with OCL type II and PF from type I and type II OC, the percentage of cells expressing CD1a, i.e., a marker of immature DCs, was significantly reduced, compared with the control. These results suggest that the OC microenvironment is enriched with factors inhibiting monocyte differentiation towards DCs. This is in agreement with the results of Chen et al., who observed that ovarian cancer ascites and supernatants from ovarian cancer cell cultures induced mature DCs to differentiate into macrophage-like cells with a CD14^+^CD1a^−^ phenotype [16]. In fact, PF in OC patients creates a complex and highly pro-tumor microenvironment with elevated levels of IL-6, IL-8, IL-10, IL-15, IP-10, MCP-1, MIP-1β, and VEGF [17]. Our findings showed that the observed effects were present in both types of ovarian cancer. However, the results obtained from type II OC patients were compelling (*p* = 0.03). This is in agreement with the study by Yang et al., who detected that mice immunized with OCL-DCs exhibited a less protective anti-tumor effect than those immunized with DCs alone. The authors conclude that DCs affected by the tumor microenvironment may promote tumor angiogenesis, which likely contributed to the limited efficacy of the OCL-DC vaccine in mice [18]. However, as shown in animal studies, the course of the differentiation of monocytes in vitro may also be altered by a previous in vivo exposure to different cytokines [19].

It has been described recently that OCL can regulate the differentiation of nonpolarized M0 macrophages by either up-regulation of scavenger receptors [SR-A] (type I) or promotion of tolerance through IL-10 and G-CSF (type II) [20]. Macrophages are recruited into the tumor microenvironment from the circulation, and they are usually polarized towards the M2 phenotype [21]. Hageman et al. reported that OC cells can polarize macrophages toward a tumor-associated phenotype in a cytokine- and chemokine-related manner in vitro [22]. Moreover, macrophages and monocytes from OC ascites suppressed proliferative response of T cells by releasing IL-10 and TGF-β [23]. This is coherent with our previous studies, where we detected elevated levels of IL-10 and TGF-β in PF of OC patients [24]. Here, we have demonstrated higher production of IL-10 in Mo-DC cultures generated from type II OC compared with Mo-DCs generated from type I OC. Our unpublished data show higher levels of IL-10 in type II OC ascites compared with peritoneal fluid from type I OC. Nowak et al. demonstrated that type II OC cells were able to release higher levels of IL-10 than type I tumor cells [25]. Together with our findings, this confirms that IL-10 is a key cytokine involved in immune tolerance development in type II OC. The high immunosuppressive activity of IL-10 may partially explain the higher pro-tumor activity of type II OC cells and confirm different progression mechanisms underlying in type I and type II ovarian cancer. Moreover, this may imply that the neutralizing effects of IL-10 may be more beneficial in the immunotherapy of patients with type II ovarian cancer.

In the present paper, the percentage of OCL type I and type II-pulsed DCs expressing co-stimulatory molecule CD86 was significantly higher in relation to the control. This proves that OCL from either type I or type II ovarian cancer can be used as a source of tumor antigens. However, the immunosuppressive OC milieu is a strong inhibitor of the DC maturation process. In fact, in this study, the PF from type I OC patients significantly reduced the percentage of cells expressing CD86/HLA-DR antigens. On the other hand, the PF from type II OC patients significantly reduced the percentage of cells co-expressing CD80/CD86/HLA-DR antigens. This may confirm our hypothesis that there are different types of immune suppression mechanisms active in type I and type II ovarian cancer. Thus, the ovarian cancer microenvironment can profoundly affect the nature of DCs, surface marker expression, and secretory function.

Due to the variety of immune-tolerant cells, we observe a failure of an effective immune response in cancer patients. Tregs are a main population of cells controlling the balance between immune response and immune tolerance. However, mechanisms responsible for differentiation of Tregs in cancer patients are not fully understood. Few papers report the increased percentage of Tregs in the peripheral blood and PF of ovarian cancer patients [12, 14] and its negative prognostic value [12, 13]. Furthermore, an increase in circulating Tregs has been observed in cancer patients treated with DC vaccines [26]. The fact that immature DCs can induce differentiation of Tregs has been known for some time, but the same property observed for mature DCs brings serious implications for immunotherapy [27]. What is interesting is that, in our studies, we have observed an increased percentage of CD4^+^CD25^high^/FoxP3^+^ Tregs both in the co-cultures of lymphocytes with autologous DCs loaded with OCL and in the cultures of lymphocytes stimulated with PF. However, while an increased percentage of Tregs was detected in the co-cultures of DCs with autologous lymphocytes in type I OC, in type II OC, significantly higher Treg percentage was observed in the cultures generated with PF. Several studies on DC vaccines in a variety of cancers have reported that DCs can induce differentiation of Tregs. Ramos et al. reported that Mo-DCs co-cultured with autologous lymphocytes from breast cancer patients induced higher occurrence of Tregs, compared with healthy donors [28]. In melanoma patients, DCs induced Treg differentiation in vitro most effectively at treatment with inflammatory cytokines. Similarly, lung cancer cell lysates promoted Mo-DCs to induce Treg differentiation in a TGF-β-related manner in vitro [29]. Also supernatants obtained from OC cell line SKOV3 were able to convert CD4^+^CD25^−^ into CD4^+^CD25^+^ regulatory cells and the neutralization of TGF-β could eliminate that transformation [30]. However, as mentioned previously, the cytokine and chemokine profile in the OC microenvironment is highly disturbed and TGF-β may be only one of the possible causes of Treg formation.

Our findings suggest that differentiation of Tregs may be induced by both the factors present in the PF and the DCs loaded with OC cell lysates. However, the type of OC may be a key to understanding what mechanisms are involved in this process. This crucial observation may shed new light on the difficulties encountered while evolving an effective response to DC vaccination in OC patients. Triggering proper maturation of DCs is a key to development of efficient and adequate response to DC vaccine in cancer patients. The elaborate network of relations between cancer cells, DCs, immunosuppressive cells, and cytokines is so far the greatest difficulty to do so. Therefore, we believe that our study may contribute to better understanding of these mechanisms and development of a closer model of the interrelations in the ovarian cancer milieu. On the other hand, our results point at inhibition of DC-induced Treg expansion as a second main area of exploration in the research on development of the DC vaccine.
